# First confirmed case of chronic traumatic encephalopathy in a professional bull rider

**DOI:** 10.1007/s00401-017-1801-z

**Published:** 2017-12-28

**Authors:** C. Dirk Keene, Caitlin S. Latimer, Lisa M. Steele, Christine L. Mac Donald

**Affiliations:** 1 Department of Pathology, University of Washington, Seattle, WA USA; 2 Department of Pathology, Royal Inland Hospital, Kamloops, BC Canada; 3 Department of Neurological Surgery, University of Washington, 352 9th Ave, Box 959924, Seattle, WA 98104 USA

There is increased concern regarding the prevalence of chronic traumatic encephalopathy [5] following repeated head impact exposure in a variety of sports [2, 4, 6, 7] and the military [3] but the existence in other populations is unknown. We present the first-confirmed case of a professional bull rider with CTE. Following sustainment of at least 15 head injuries over a 10-year period confirmed by collateral sources, records review, and ante-mortem imaging studies, the majority witnessed and documented on videotape, he committed suicide. Unique to this case, we performed imaging-guided brain tissue sampling for neuropathological investigation. This approach may provide for more focused tissue sampling that is sensitive and flexible to the heterogeneity of brain injury complementing standard neuropathological evaluation strategies.

Past medical history identified first-diagnosed concussion at age 16 with confirmed loss of consciousness (LOC) and additional exposures approximately biennially until age 21 when he sustained five head injuries over the course of a 12-month period. Each incident involved LOC for minutes followed by disorientation, confusion, ocular disturbance including photophobia, and periods of anterograde amnesia lasting hours to days. The decedent was evaluated by onsite medical personnel and/or admitted to a hospital for observation with radiographic CT examination, noted as unremarkable each time. At age 23, he sustained a blow to the head after being stepped on by a rearing bull crushing his helmet with LOC for 1 h, meeting criteria for moderate brain injury [8]. Initial Glasgow Coma Scale (GCS) at hospital presentation was 10, and remained 10 for 24 h before returning to 14 out of 15 by day 2. CT evaluation again was negative for pathoanatomical brain injury lesions. MRI evaluation completed 3 months later identified multiple regions of hemorrhagic foci bilaterally in the frontal lobes, right temporal lobe, left hippocampus, and left brainstem, consistent with microhemorrhage following shear injury meeting radiographic criteria for diffuse axonal injury [1].

Following these exposures, the decedent was noted to have post-traumatic headache, memory loss, concentration problems, attentional dysfunction, mood lability, disinhibition, diplopia, photophobia, phonophobia, vestibular dysfunction, insomnia, irritability, explosivity, depression, anxiety, dysarthria with mild aphasia, difficulty with mental flexibility and planning, motor slowing, exaggerated somatic concern, hostility, and conceptual disorganization. Family members described a very bright, jovial, and affable young man who was conscientious and loving but in the last 6–9 months of life rapidly deteriorated, becoming reclusive and hypervigilant with paralyzing panic attacks, and displaying significant behavioral changes characterized by erratic and impulsive decisions until his death at 25.

Following consent for brain donation, familial consent was provided for review of medical records, clinical CT, and MRI scans collected on the decedent prior to death in accordance with regulations. The decedent’s fixed brain was examined for gross findings and then imaged ex vivo with high-resolution MRI for co-registration to the ante-mortem scans. This allowed imaged-guided tissue sectioning for pathoanatomic lesions visible on the ante-mortem MRI that may be grossly unremarkable. Standard sampling was performed to evaluate for traumatic brain injury, CTE [5], and other neurodegenerative processes. Sampled regions included bilateral superior and middle frontal gyri, orbitofrontal cortex, superior and middle temporal gyri, anterior temporal lobes, inferior parietal lobules, hippocampi and entorhinal cortex at two levels, amygdalae, thalami, hypothalami, basal ganglia with internal capsule, midbrain with substantia nigra, pons with locus coeruleus, medulla with dorsal motor nucleus of the vagus nerve, and cerebellar cortices with dentate nucleus. Additional samples of bilateral frontal and parietal white matter were submitted based on neuroimaging guidance.

All tissue sections were stained with hematoxylin and eosin/luxol fast blue (H&E/LFB). Glial fibrillary acidic protein (GFAP, reactive astrogliosis), ionized calcium-binding adapter molecule1 (Iba1, microglial activation), paired helical filament (PHF) tau, TDP43 (ubiquitin-positive-tau-negative inclusions), alpha synuclein, amyloid (Aβ) and Bielschowsky stains were performed on select regions. The fresh whole brain weighed 1360 g and external examination was normal for age with no evidence of mass lesions, destructive lesions, hemorrhage, herniations, or cortical atrophy. There was a small cavum septum pellucidum without lateral ventricular enlargement. Definite surface contusions, other cortical lesions, cavitary lesions, and cerebellar lesions were not identified. Subcortical white matter was full and firm. Deep cerebral nuclei, and brainstem white matter and nuclei were symmetric and well formed. The brain tissue slabs were aligned with the imaging for MRI-guided lesion sampling supplemented with standard neuropathological sectioning generating 42 samples for staining.

**Fig. 1:**
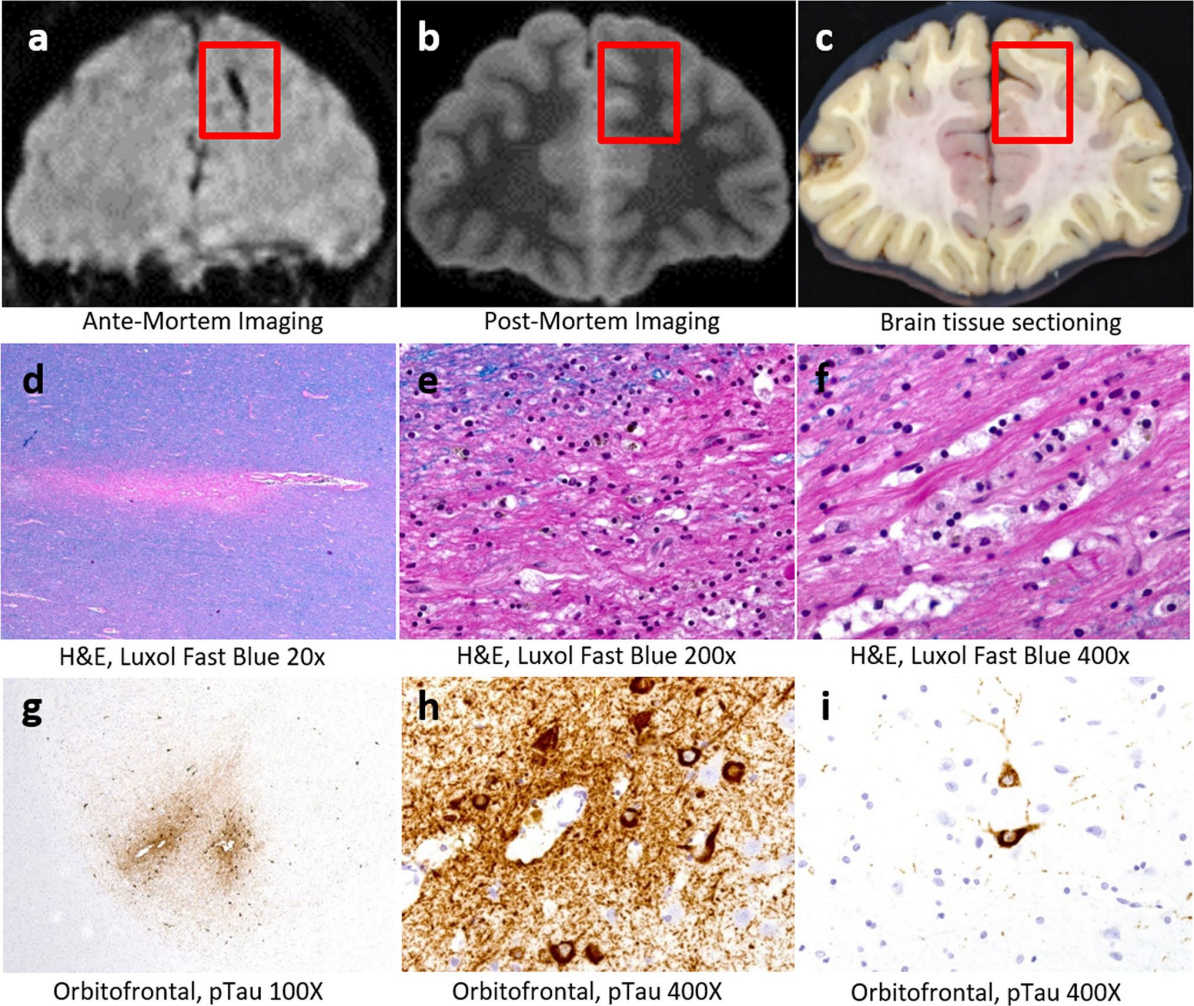
Ante-mortem imaging (**a**) was co-registered with post-mortem imaging (**b**) for image-guided tissue sectioning (**c**). In normal-appearing regions, image-guided sectioning identified focal chronic axonal injury including axon loss (**d**), hemosiderin pigment deposition (**e**) and foamy macrophages (**f**). Positive phospho-tau immunostaining of neurofibrillary tangles, neurites, and glial inclusions was present in bilateral orbitofrontal cortex with deposition localized perivascularly in sulcal depths (**g**–**i**)

A microscopic contusion was identified in the left medial temporal lobe characterized by dense superficial reactive astrocytes and focal hemosiderin deposition. Multifocal microscopic frontal white matter lesions characterized by remote perivascular hemorrhage (hemosiderin) associated with organizing axonal necrosis (axon loss associated with foamy macrophages) were present in MRI-guided samples indicating remote axonal injury. These lesions were not visible grossly but were identified by image-guided sectioning (Fig. 1a–f). Examination for neuropathologic changes of frontotemporal lobar degeneration, Lewy body disease, Alzheimer’s disease, and other progressive degenerative processes was negative.

Microscopic examination revealed diagnostic evidence of chronic traumatic brain injury, including CTE lesions, a remote contusion in the left medial temporal lobe and focal chronic axonal injury. Specifically, the pathognomonic lesions of CTE (depth of sulcus perivascular glial and neuronal tau) were identified in the bilateral orbitofrontal cortex (Fig. 1g–i). Although pTau-positive neurites and rare neurons were identified in frontal, cingulate, limbic structures, and parietal and temporal cortex, pathognomonic CTE lesions were not identified in these regions. While CTE pathology stages have been proposed [5] they are neither fully validated nor well accepted; nevertheless, the distribution and burden of pathological tau was most consistent with low-stage CTE pathology. The contrast between high-exposure history and low-stage CTE may be due to the young age at death given that it is thought the tau pathology often develops over years after exposure.

Chronic traumatic encephalopathy was identified for the first time in a professional bull rider. It appears repeated exposures caused brain injury contributing to altered mental health behavior and untimely death. CTE lesions were only identified in orbitofrontal cortices, while in American football and other sports these lesions are often identified in superior and lateral cortical frontal and temporal sulci. Further studies are needed to determine whether neuropathological patterns of injury differ between bull riding in which the impact mechanics are dictated a 2000 lb bull and other neurotrauma exposures from sports which are more dictated by human size and whether lesion distribution impacts clinical penetrance and phenotype. Additionally, image-guided tissue sectioning combining ante-mortem MRI exams co-registered with post-mortem MRI enhanced selection of brain tissue for analysis and may present a strategic approach to pathological investigation of heterogeneous brain injuries.
